# Adolescent lives matter: preventing HIV in adolescents

**DOI:** 10.1097/COH.0000000000000453

**Published:** 2018-04-04

**Authors:** Audrey Pettifor, Marie Stoner, Carey Pike, Linda-Gail Bekker

**Affiliations:** a University of North Carolina, Chapel Hill, North Carolina, USA; b The Desmond Tutu HIV Centre, University of Cape Town, Cape town, South Africa

**Keywords:** combination prevention, HIV, youth

## Abstract

**Purpose of review:**

Many of the almost 2 million HIV infections that occurred globally in the last year occurred among adolescents and young people, particularly those from East and Southern Africa and within key populations. Global HIV epidemic control will require that new infections among these youth populations be curtailed. This review examines the most effective prevention approaches to reach these adolescent populations in the next 5 years.

**Recent findings:**

Adolescents are in transition and are developmentally unique. They have specific needs and challenges, which if not addressed will result in less than successful interventions. Tailored, layered, combination prevention packages that take into account specific adolescent needs and involve biomedical, behavioural and structural components are recommended. These packages should be designed for and with the meaningful input of adolescents, and involve their peers in their implementation and execution. Where possible, age-appropriate health and social interventions that go beyond HIV should be bundled and offered in a variety of community-based venues that are already acceptable to and frequented by adolescents.

**Summary:**

It is urgent that we reach adolescents globally with the most effective HIV prevention approaches. HIV prevention investment in this population has immediate and longer-term benefits.

## INTRODUCTION

Adolescents are disproportionately affected by HIV worldwide, and yet are rarely specifically prioritized in national HIV-plans and programming efforts. In 2016, 260 000 adolescents between 15 and 24 years were infected with HIV, with 44% more infections amongst young women than their male counterparts [1,2].

Within the larger at-risk population of adolescents, there are specific concentrated groups that are particularly vulnerable. Young women outside of the school system are isolated and unsupported by the networks and structures that could contribute to healthy development and reduce their vulnerability. Their vulnerabilities often stem from family economic imperatives, teenage pregnancy or a lower social priority to keep young women in school [3^▪▪^,4^▪^]. In addition, young key populations throughout the world carry a high burden of HIV and increased risk [5^▪^]. These key populations include young MSM, transgender youth, young people who inject drugs and young sex workers as well as young people who find themselves on the wrong side of the law [5^▪^]. Although large-scale interventions are needed when combating a generalized epidemic, a targeted and focused approach that caters to these key populations is required to efficiently and successfully prevent new infections [6].

HIV prevention for adolescents is particularly critical in a world where the youth population is rapidly expanding. Over half the population of the world is currently under the age of 30 and the majority of these young people reside in developing countries; it is predicted that this proportion of youth will increase markedly in the next 20–30 years [7]. Beyond HIV, almost 35% of the global burden of disease has its roots in adolescence [8]. Adolescence is a critical transition period for adolescents, as well as a critical investment period, where rewards of prevention efforts can be significantly multiplied across health and sociostructural domains. Described as the triple dividend, meaningful investment in prevention in this age group is likely to impact both the immediate and longer-term horizons [8].

Adolescence represents a transition from puberty to independence and self-sufficiency [8]. During adolescence, there are significant biological, psychological and behavioural developments, which occur alongside busy schedules and explorative learning. Adolescent brain maturation looks to the satisfaction of immediate needs and mitigation of short-term dangers [9]. As a result, decisions around health tend to be more reactionary than preventive, as reflected in the rates of sexually transmitted diseases (including HIV), which are highest amongst adolescents aged 15–24 years [10]. In addition, there are high rates of unintended pregnancy, contraceptive need and failure in adolescents, with child birth being the second leading cause of death amongst 15 to 19-year-old girls in developing countries [11–13].

Helping youth to prioritize health and engage in services is challenging when prior experience in healthcare interaction is limited, and skeptism of confidential health services is high [14]. HIV-related deaths are rising among adolescents globally, despite their decline across all other age groups [15]. In regions such as Sub-Saharan Africa (SSA), young women and girls remain most vulnerable, with AIDS the number four cause of death [16]. In the face of other health challenges such as mental health, substance abuse and pregnancy, adolescents may see HIV/AIDS as a low concern even in high-risk communities [17]. Therefore, prevention efforts should be tailored to this population, taking into account their unique challenges, strengths and opportunities to ensure successful engagement and outcomes. 

**Box 1:**
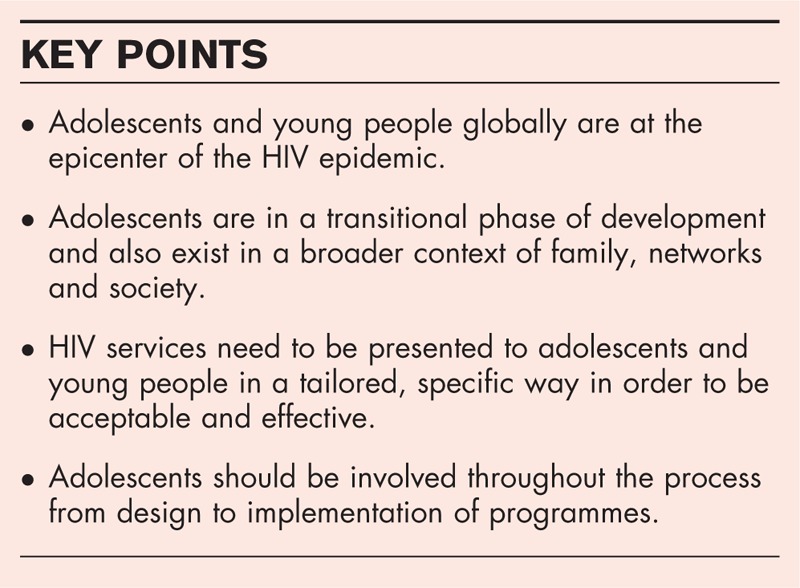
no caption available

## THE ADOLESCENT-FRIENDLY APPROACH TO PREVENTION

**FIGURE 1:**
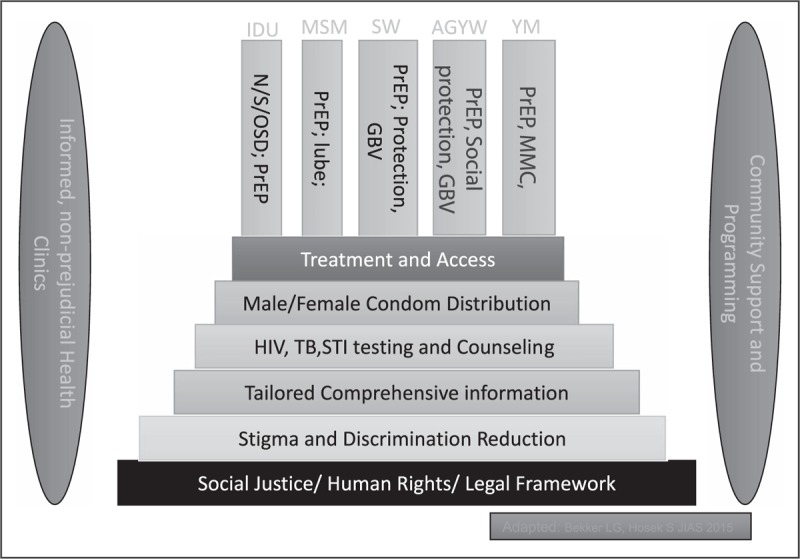
An illustration of the multiple layers of interventions required to cater to populations with varying risk factors. AGYW, adolescent girls and young women; GBV, gender-based violence; MMC, medical male circumcision; N/S/OSD, needle exchange and opioid substitution drugs; PrEP, pre-exposure prophylaxis; SW, sex worker; YM, young men; Based on [5^▪^].

It is now widely acknowledged that combination HIV prevention packages that are effective, acceptable, scalable and address multiple key risk factors or avenues for HIV transmission have the greatest combined impact [6,18]. These programmes are particularly advisable for adolescents whether young women, MSM or IDU and also need to be tailored to the adolescent's needs rather than around a specific intervention [5^▪^]. Multiple layers of interventions are required to cater to multiple levels of risk, which arise from different influencing factors (Fig. 1) [5^▪^].

## THE SOCIOECOLOGICAL MODEL

**FIGURE 2:**
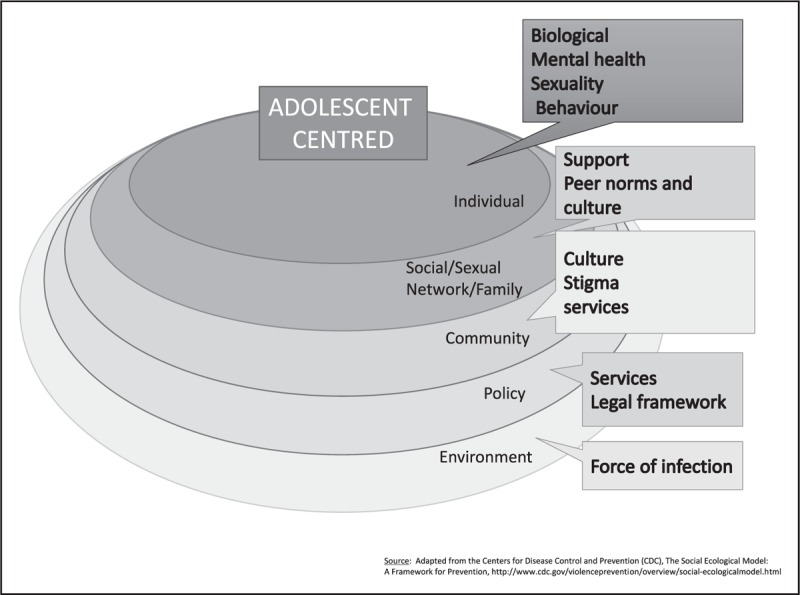
A socioecological framework for HIV prevention amongst adolescents. Based on [19].

A time of identity formation, adolescence, is also about experimenting with separation from significant adults and an increasing sense of independence [8]. Therefore, programming for adolescents should acknowledge the importance of the individual and their needs while taking into consideration their peers, sexual and social networks, families, and the social and cultural context in which the adolescent exists. Each of these influences represent key areas wherein interventions could intersect with adolescent life. Finding ways to build and bundle age appropriate prevention interventions with other promotive health opportunities in consultation with youth and offering services in places wherein youth are more likely to congregate may have a higher chance of uptake than more conventional approaches (Fig. 2) [5^▪^,^14^,^19^].

Adolescents are faced with constant choices about what to engage with whilst also formulating lifelong beliefs and ideas. It is not only a time of experimentation, when errors are common but also a time where knowledge can be gained quickly if relevant comprehensive information within a harm reduction paradigm are offered [20,21]. Individual engagement with healthcare services, such as contraception and HIV, are governed by knowledge, exposure to health promotion and perception of HIV risk and their own ability to plan and predict sexual encounters (not always expected or easy at this age).

Although this is a time when independence, privacy and the ability to discreetly initiate and use prevention interventions may be highly regarded, the adolescent's ability to adhere to daily programmes or biomedical interventions such as oral pre-exposure prophylaxis (PrEP) can be limited. Although not yet proven, longer-term, less frequent biomedical interventions (such as injectable PrEP), when provided in the context of adolescent-friendly services, may be a better solution for this population, as evidenced in long-acting, reversible contraception (LARC). However, there are other behavioural and structural risk factors that could challenge uptake and adherence to some prevention methods, especially associated with relationships with large sex–power imbalances. Here, more discreet, user-controlled methods, for example, Vaginal micobicide rings, may be preferred [12,13].

## NETWORKS AND RELATIONSHIPS

Young women who have age-disparate relationships are more likely be exposed to infection due to the higher prevalence of HIV in older men. Age-disparate relationships do appear to contribute to the high risk of HIV in young women [22,23,24^▪^,^25^–^29^], but the association may vary depending on the age of the woman and the severity of the epidemic [30,31]. A similar increased risk exists for young men experimenting with same sex relationships who may have their first relationships with older MSM. Transactional sex has been consistently associated with HIV infection, but only one study has used a measure of incident rather than prevalent, infection limiting conclusions based on current evidence [32,33,34^▪^,^35^]. The nature and motivations underlying transactional sex are varied and can include agency, social mobility, sex inequality, love, masculinity, poverty, survival and helplessness [36,37]. Interventions should be tailored to different underlying motivations and should consider young women's agency in the relationship. Although sex inequality is central to all interventions, those focusing on poverty alleviation will work only in some contexts, while programmes that address self-esteem and future aspirations will be more effective for motivations related to social status and peer approval [36].

The economic and social pressures that result in young women having transactional sex or older partners also contribute to unequal power dynamics within a relationship. Unequal power dynamics can increase risk of HIV infection by undermining other sexual behaviours such as condom use and increasing the potential for intimate partner violence (IPV) within a relationship [38–40]. IPV has been associated with an increased risk of HIV through risky sexual behaviours such as unprotected sex and through forced sex [41,42]. Interventions to address norms about sex inequality have been shown to have an effect on attitudes related to violence, experience of violence and other factors associated with risk of HIV [43,44].

The influence of peers is critical for this age group, wherein risky, impulsive behaviours are enhanced in the presence of peers [45]. Practically, youth engagement involves having adolescent representatives involved in the clinical trial design process, youth community advisory boards, peer navigators in clinical settings and youth-lead advocacy campaigns [46]. Using modern technology to enhance this through applications, gaming and other social media platforms can also take advantage to improve buy-in and engagement [47].

## FAMILY AND PARENTS

At the household level, families provide the primary support structure in which young people grow, develop and transition into adulthood [3^▪▪^]. Due to changing marriage dynamics, parental death from HIV, parental migration for work and other factors, there has been an increase in single parent families and households where young people live with extended family members rather than parents [3^▪▪^]. Orphan status and family instability have been linked to poorer health outcomes for adolescents, including risky sexual behaviours, early pregnancy and risk of HIV and other sexually transmitted infections [48–50]. Family instability may contribute to sexual risk because puberty is a time where parental responses and supervision are critical for shaping how adolescents respond to new experiences. Dysfunctional family norms that promote sex inequity and violence or exposure to violence can result in substance abuse, school dropout and other mental health issues for young women [3^▪▪^]. Conversely, parent/guardian models of positive responses and teachings around sexuality and HIV prevention are important for promoting HIV prevention and well tolerated sex, particularly in younger adolescents who still rely heavily on parental guidance and care. Family based HIV-prevention programmes have been shown to increase safer sexual behaviours and to improve adherence and other behaviours in HIV-positive youth [51–54]. However, more research is needed to fully understand how families can be supported and encouraged to promote healthy behaviours in adolescents.

Although some young people may experiment sexually, as they develop a mature identity, for sexual minority youth, coming out is not a phase but an important part of their life. Sensitive discussions about feelings of anxiety, fears and internalized stigma may be helpful. Coming out for many young MSM can also mean risking rejection and even the loss of family support. As young people are less likely to have the resources to support themselves, this can lead to considerable hardship (e.g. homelessness, mental health problems and substance abuse). Local laws and general attitudes will influence a young person's decision about whether they are able to come out to fully express their identity and desires, especially in countries where sexual and gender minorities face discriminatory laws [55]. Because adolescence is a critical period in identity formation, adverse experiences impair further psychosocial development and decisions around safer sex practices. Support from other key adult role models or significant peers can be very useful in these settings. Technology and social media platforms can also provide new solutions, as there are innovative eHealth interventions aimed at overcoming social isolation to reach and connect young MSM [56,57].

## STRUCTURAL AND COMMUNITY FACTORS

Poverty is an important structural driver of adolescent sexual risk, particularly for women, as it creates an environment conducive to transactional relationships and makes it difficult for girls to remain in school due to inability to pay school fees, purchase uniforms or detraction from income-generating activities. Failure to remain in school is a strong predictor of adverse sexual health outcomes, with less educated women being at a higher risk for HIV and early pregnancy. Conversely, education, including both increased level of education [58–64] and school enrolment among adolescent girls, has been shown to prevent HIV infection [22,65–68]. This may be because schools create a ‘safe space’ and occupy women's time, such that young women have fewer older partners, fewer partners overall and safer sexual behaviour [69,70]. ‘Safe space’ interventions are typically girl peer support groups that meet in a safe, physical space, and promote a strong social network [71,72]. This empowers young women by providing them with resources and a stronger network of peers to aid in decision-making. Similar opportunities and safe spaces can be set up for young same sex individuals especially in communities wherein same sex activities are illegal or stigmatized [73].

Social protections, including cash transfers, are a promising tool for reducing poverty, keeping girls in school, reducing risky sexual behaviour and ultimately preventing HIV. Social protection can encompass an array of interventions and may involve cash paid to a young woman, her guardian or both; may be conditional or unconditional; and may be paid in cash or in kind, such as in the form of waived school fee. In a review, nine of 10 studies that involved cash transfers had a positive influence on HIV-related behaviours [74]. Two randomized controlled trials in South Africa investigated the impact of cash transfers for adolescent girls on HIV risk. Neither showed any impact on HIV incidence; however, HPTN 068 showed reductions in experiences of physical violence from partners and some sex behaviours and the CAPRISA trial showed a reduction in herpes simplex virus - type 2 [75^▪^,76^▪^]. There is some evidence from other settings that cash transfer interventions may reduce transactional sex, frequency of sex and partner age-disparity; however, reports of their effect on HIV incidence remain inconsistent [76^▪^,^77^,^78^].

Within communities, sex inequality and sex norms that condone violence against women, or sexual minorities and promote traditional sex expectations can increase risk of HIV. Supporting a sexual and reproductive life free from violence and abuse is an important end in itself and may impact HIV prevention outcomes. Empowerment-based interventions that involve a participatory process and address sex norms directly present a potential solution. Three such programmes in sub-Saharan Africa have shown reduced IPV and related outcomes; however, these interventions are typically multisession and resource-intensive [43,44,79]. Synthesizing the most effective elements of these interventions and delivering elements of them in fewer or more diverse sessions could enhance impact. Community mobilization programmes have also been effective in shifting community sex norms and attitudes that underlie violence against women and risk of HIV [44].

There is evidence that the well being of a community is associated with young women's sexual health, where women who perceive their communities as cohesive and supportive are less likely to engage in condomless sex, have reduced rates of sexually transmitted infections (STIs) and delay sexual debut [80–85]. Conversely, stigma surrounding sexuality, sexual risk (promiscuity and age of debut) and HIV results in reduced HIV disclosure and increased risk of secondary infection. Stigma is often most keenly felt by adolescents and can stop them from seeking information and health services, leading to riskier behaviours and more infections. Attitudes should be grounded in acceptance, harm reduction, normalization of HIV prevention and services, and laws that underpin public health and health rights.

## BIOMEDICAL INTERVENTIONS

Adolescents who receive both behavioural and structural support also seek health knowledge and uptake of and demand for appropriate clinic services and biomedical prevention options; however, work is needed to improve the supply of health services that are adolescent friendly and accessible to this population [5^▪^]. Adolescent-friendly services (AFYS) offer healthcare at convenient times and spaces for adolescents; they address adolescent sensitivities and needs for privacy, and carry a de-stigmatized, harm reduction approach to adolescent sexual engagement. [14] Adaption of the healthcare community to this service approach may include a variety of models from stand-alone multifunctional youth facilities, to mobile services and pop up services in other youth friendly venues. AFYS alone will never be a sufficient draw card, and for this reason, integration of HIV healthcare with other STIs, family planning and diverse issues of mental health and general wellbeing including life skills and empowerment should be included [5^▪^,^86^].

Biomedical HIV prevention is a rapidly growing field, and now many countries offer a standard of prevention that includes oral PrEP, post-exposure prophylaxis, medical male circumcision, testing including self-testing, harm reduction for drug users, STI screening, antiretroviral therapy for pregnant women and positive partners and of course condoms. Notwithstanding the ethicolegal challenges, new biomedical interventions should include and involve adolescents as soon possible to ensure these new modalities are incorporated into any platforms as soon as they become available to adults [87]. Microbicides in the form of the Dapivirine ring have been found to be effective if used but are not yet commercially available, while injectable PrEP and implants are moving up the pipeline [88^▪^]. Medical male circumcision (MMC) reduces risk of HIV acquisition among HIV-negative men. Promoted most widely in SSA, uptake has been higher in some countries than others [90]. As a once off, relatively low medical risk procedure, the benefits of MMC accrue over many years making this a highly cost-effective intervention [91]. PrEP and microbicides have been reported to be acceptable to adolescents in many settings especially with the promise of wider administration and dosing frequency choices. Far fewer studies have been conducted to demonstrate efficacy or feasibility, but most recently, demonstration studies of oral PrEP in adolescents are being reported, whilst enthusiastically taken up, daily oral PrEP poses challenges of daily dosing fatigue, perceptions of stigma and worries about side effects [86,88^▪^,^89^].

Many lessons can be taken from contraceptive use in adolescents. There is increased recognition that tailored programmes provide more support both for understanding and persistence. Because youth are generally well, there is a greater need to provide a sex and health positive message and reduce the medicalization of prevention for adolescents. Effective demand creation strategies and public awareness campaigns are those that make use of gain-frame messaging.

Recognizing that these interventions may only be needed for ‘seasons of risk’, many countries are grappling with exactly which populations should be offered oral PrEP. Numbers at risk are large, particularly in countries and regions where there is a competing significant treatment burden. Young key populations globally and young women and girls, particularly in East and Southern Africa, remain a compelling group [88^▪^,^89^]. Understanding how to provide novel biomedical interventions such as oral PrEP within acceptable, scalable platforms, with relevant and compelling demand creation, and effective adherence support whilst addressing other key structural and behavioural components are urgent implementation science questions.

## POLICY IMPLICATIONS

Evidence presented here leads us to make the recommendations listed in Table 1.

**Table 1:** Policy implications

HIV prevention services for adolescents should be specific, tailored and comprehensive taking into account this age group's unique development stage and needs.
There should be frank and intentional inclusion of adolescents in design, implementation and execution of programmes.
HIV services should be offered as part of a broad programme on sexuality, sexual and reproductive health with a public health and individual health rights lens.
Adolescent-friendly services should be age-bundled and offered in adolescent-appropriate venues, which are preferably ‘walkable’ and community based.
Social media and other innovations can inform, create demand and help monitor uptake and use of services.
Adolescents should be involved in clinical development of new modalities sooner rather than later
Investment in this age group should be made recognizing the future dividend in healthy adults and healthy parents.

## CONCLUSION

HIV brings adolescent health into sharp focus globally and offers an opportunity to address the health and wellbeing of this important and growing population. HIV prevention must be prioritized in young populations with a high incidence and specifically in young key populations who are particularly vulnerable throughout the world. Meaningful combinations of prevention interventions will be most effective where they are tailored according to geographical and adolescent population needs. Implementation needs to be effectively monitored and impact carefully measured. Country programmes such as ‘She Conquers” in South Africa and the PEPFAR supported DREAMS initiative in Southern and Eastern Africa will be the first generation of evidence for adolescent focused, tailored and layered approaches targeting young women [92,93]. There is a need for additional similar programmes in diverse adolescent populations around the world.

## Acknowledgements

LGB has received honoraria from Merck and Jansen and her organization has received donations from Gilead for PrEP demonstrations. The remaining authors have no conflicts of interest.

### Financial support and sponsorship

This work was supported by the HPTN. Overall support for the HIV Prevention Trials Network (HPTN) is provided by the National Institute of Allergy and Infectious Diseases (NIAID) of the National Institutes of Health (NIH) under Award Numbers UM1AI068619 (HPTN Leadership and Operations Center), UM1AI068617 (HPTN Statistical and Data Management Center), and UM1AI068613 (HPTN Laboratory Center). The content is solely the responsibility of the authors and does not necessarily represent the official views of the National Institute of Allergy and Infectious Diseases or the National Institutes of Health.

### Conflicts of interest

*None*.

## REFERENCES AND RECOMMENDED READING

Papers of particular interest, published within the annual period of review, have been highlighted as:

▪ of special interest
▪▪ of outstanding interest
